# Reasons behind Low Cervical Screening Uptake among South Asian Immigrant Women: A Qualitative Exploration

**DOI:** 10.3390/ijerph19031527

**Published:** 2022-01-28

**Authors:** Zufishan Alam, Hanoor Deol, Judith Ann Dean, Monika Janda

**Affiliations:** 1 Centre for Health Services Research, Faculty of Medicine, Princess Alexandra Hospital Campus, The University of Queensland, Woolloongabba, QLD 4102, Australia; m.janda@uq.edu.au; 2 Faculty of Medicine, Herston Campus, The University of Queensland, Herston, QLD 4006, Australia; hanoor.deol@gmail.com; 3 School of Public Health, Faculty of Medicine, Herston Campus, The University of Queensland, Herston, QLD 4006, Australia; j.dean4@uq.edu.au

**Keywords:** cervical cancer, screening, HPV test, self-sampling, immigrant, South Asian

## Abstract

Despite advancements in prevention strategies, cervical cancer remains a leading cause of death among underprivileged women. Although Australia has low age-standardized cervical cancer incidence rates compared with other countries, disparities exist in cervical screening uptake among certain population subgroups, especially those from culturally and linguistically diverse (CALD) backgrounds. South Asian immigrant women have been reported to have lower cervical screening uptake than Australian-born women and those from other immigrant backgrounds. The objective of this study was to gain insight into the reasons and barriers for low cervical screening participation among South Asian immigrant women, through qualitative exploration. Semi-structured, in-depth interviews were conducted with 20 women, aged 26–50 years, living in Queensland, Australia, who were recruited via purposive sampling. After translation and transcription of recorded interviews, data was analysed via inductive thematic approach. Resulting themes, illustrating barriers towards screening, included: lack of cervical cancer and screening knowledge, especially of the changes in the revised screening program; effect of preventive, health-seeking behaviours; health care system factors; role of practical constraints and influence of sociocultural beliefs. Results suggest that culturally informed interventions, that involve relevant information provision and behavioural change strategies, to clarify women’s misconceptions, are required.

## 1. Introduction

With the recent launch of a global initiative to eradicate cervical cancer in the near future by the WHO [1], disparities in cervical cancer prevention between countries, and among population subgroups within countries, based on differences in socioeconomic status, health care system, and culture have come to the forefront [2,3,4]. In Australia, cervical screening tests are offered to women and people with a cervix, aged 25–74 years, every 5 years. However, cervical screening uptake has been reported to be low among Aboriginal and Torres Strait Islander (First Nation) peoples [5] and across culturally and linguistically diverse (CALD) groups with immigrant backgrounds [6]. With globalization leading to increased immigration, nearly 30% of the Australian population comprises immigrants. Over the past decade, the number of people migrating from South Asian regions (India, Pakistan, Bangladesh, Sri Lanka, Nepal, Bhutan, and Maldives) to Australia has increased significantly [7]. Studies assessing cervical screening uptake in Australia suggest that women born in South Asia tend to have lower cervical screening uptake than those born in Australia and other regions of the world (odds ratio: 0.54; 95% CI: 0.48–0.61) [8,9]

Uptake of preventive health services is often the result of a complex intersection of individual, interpersonal, and environmental level factors [10], with sociocultural determinants having particular importance for uptake of sexual and reproductive health services [11,12]. Studies show that numerous traditional and ethnic barriers are responsible for lower cervical screening uptake among immigrants compared with the general population [13,14,15]. Qualitative studies show that Australian women with an immigrant background lack conceptual knowledge regarding cervical screening, have difficulties navigating the health care system, and face language barriers [6]. Modesty has also been reported as a common barrier in seeking cervical cancer preventive care among immigrant women in various studies [16,17,18]. Additionally, for many Thai women, misconceptions based on traditional beliefs associated with female body parts and menstrual blood influence screening knowledge and uptake [17]; whereas, for some African immigrant women, genital mutilation experiences account for a significant hurdle [19]. Quantitative studies indicate that socioeconomic, educational, and marital status, and duration of residence in Australia are associated with screening uptake [9,20,21]. However, evidence for cervical screening barriers for South Asian immigrant women in Australia is scarce.

In Australia, cervical screening is offered free of charge under Australia’s universal health insurance scheme (Medicare) to immigrants that are permanent residents [22]. The National Cervical Screening Program was renewed in 2017 and the Papanicolaou (Pap) test was replaced by the human papilloma virus (HPV), test as the recommended test for cervical screening [23]. Self-sampling cervical screening was also included for the first time in the renewed program for under-screened and never-screened women. The views of South Asian immigrants on the HPV test and self-sampling have not been explored; thus, this qualitative study was conducted to gain a better understanding of their perspectives on cervical cancer, its screening with the changes in the renewed program, and the barriers towards screening uptake.

## 2. Materials and Methods

### 2.1. Study Design and Context

This paper reports on the findings of the qualitative phase of a larger sequential mixed-methods study, consisting of a systematic review and a quantitative survey, results of which have been reported previously [6,24]. The systematic review critically evaluated the cervical screening practices and uptake among immigrant women in Australia, whereas the survey quantified the proportion of South Asian immigrant women that had knowledge of (53.5%) and participated (55.4%) in cervical screening, and the factors associated with the uptake. The aim of the qualitative phase, including the semi-structured interviews reported here, was to elaborate on the survey findings and to provide women who were unable to complete the survey, due to literacy or English language restrictions, with a voice. The interview questions, informed by the literature and quantitative survey results, focused on enablers and barriers to screening and steps that could be undertaken to increase participation in cervical screening.

### 2.2. Participants and Recruitment

Women were recruited using purposive and snowball sampling techniques and through organisations working with migrants [25,26]. They were recruited from different venues frequented by South Asian women, including community centres, social gatherings, and multicultural events. Women were eligible to participate in this study if they were born in any of the countries constituting South Asian ethnicity—according to the Australian standard classification of cultural and ethnic groups [27]—aged 20–75 years, residing in Queensland, Australia, and able to communicate in one of the South Asian native languages that the first author (ZA) could speak (Urdu, Hindi, Punjabi, and Fijian Indian) or English. Women with South Asian ancestry born in Australia, to parents born in South Asian countries, and those with a history of cervical cancer, were excluded. The aim was to specifically capture the views of women with lower English literacy levels, and of those that took part in the survey earlier and volunteered to shed more light on the topic. Recruitment and interviews continued until saturation of emerging themes was attained [28].

### 2.3. Data Collection

The interviews were carried out between October and December 2019, either in person at community venues that were convenient and comfortable for the participants, or via telephone. Twenty women were approached, all provided written informed consent, or, in the case of telephone interview, verbal informed consent, to participate in the study. The interviews lasted approximately 40–45 min and were audio-recorded. The interview guide consisted of open-ended questions, with prompts to help the interviewer direct the conversation and probe relevant topics. For example, to identify the barriers towards cervical screening, women were asked if they had taken the cervical screening test here in Australia, and if not, what is preventing them from getting it. Similarly, another question queried about required areas of knowledge that could increase screening participation. However, the interview guide was not strictly adhered to, as it was important to allow the participants to communicate freely. This encouraged rapport between the interviewer and interviewee, thus creating a relaxed environment.

### 2.4. Data Analysis

The interviews in South Asian languages were translated to English by the first author, followed by verbatim transcription of all interviews. To ensure fidelity, the transcripts were back-translated, and checked for accuracy by a person fluent in above mentioned languages, not otherwise involved in the data collection. Data analysis followed an inductive thematic approach [29]. This approach allowed the emergence of themes that provided greater understanding of perspectives and behavioural attitudes towards cervical cancer prevention within this community. The transcripts were analysed by two members of the research team (ZA and HD) independently. They initially read and reread the transcripts to gain familiarity with the data and to relate the emerging data with the study’s aims. NVivo software [30] was used to manage the data analysis, which allowed formation of nodes and sub nodes representing various themes and subthemes. They were then refined with similar nodes being combined and new emergent subthemes being split, thus developing a codebook. The codebooks created by the two researchers were reviewed and compared at regular meetings to create the final codebook. Any differences were resolved by mutual discussion and by involvement of other authors where required.

### 2.5. Ethical Considerations

The ethical clearance for the study was obtained from Human Research Ethics Committee, University of Queensland (Approval no: 2018001749). Each participant signed a written informed consent form prior to their individual interviews. They were informed about the confidential nature of the collated information from the interviews, and were informed that they were free to withdraw at any stage without penalty or impact on future access to services where they were recruited and/or interviewed. To ensure confidentiality, the transcribed interviews were deidentified and allocated a number, ensuring no identifying information was present. Formal consent was also collected from the administrative personnel of all the community venues used for recruitment to request their permission to conduct recruitment and data collection at or via their sites.

### 2.6. Rigor

Maximum efforts were made to capture participants’ perspectives with their original intent, both during collection and analysis. Involvement of two researchers who undertook separate analyses allowed comparison and reflection on the true meaning of the women’s views. Additionally, regular meetings with the research team were held to discuss the arising themes and amend them when necessary. Furthermore, to ensure reflexivity, the lead researcher maintained a journal throughout each step, to record personal reflections and internal feelings towards the findings.

## 3. Results

### 3.1. Participants’ Characteristics

The 20 women participating in the study were from ethnically diverse South Asian backgrounds: Indian (*n* = 9); Pakistani (*n* = 6); Bangladeshi (*n* = 2); Sri Lankan (*n* = 1); Indian Fijian (*n* = 1); Nepalese (*n* = 1). Their age ranged between 26 and 50 years (mean = 35.4 years), and they had lived in Australia for 1–20 years. Nearly half (*n* = 9) had 3 or more children (Table 1). Although all women had some form of formal education, only five had completed an undergraduate university degree, the remaining had completed secondary school education only. Of the 20 interviews conducted, 10 were carried out via telephone and 10 in person at community venues, and 15 of the 20 interviews were conducted in native South Asian languages (Urdu, Punjabi, Hindi, Fijian Indian). Twelve women reported having had a cervical screen previously, but six of this sub-group were not up to date, according to the screening guidelines.

### 3.2. Themes

Three themes were identified during the inductive thematic analysis: (1) deficits in knowledge of cervical cancer and its prevention opportunities; (2) barriers influencing cervical screening uptake; (3) increasing cervical screening participation. These themes, with identified subthemes, are presented in the following section, along with participants’ direct quotations.

**Theme 1:**  **Deficits in knowledge of cervical cancer and its prevention**

1. Patterns of cervical cancer awareness vary:

Significant lack of knowledge, and even unfamiliarity that cervical cancer exists, was evident among many participants. Many had not heard the term “cervical cancer” before and were not aware of the term used in their first languages for the disease. Several participants confused it with the respective term used for uterine cancer in the native languages such as cancer of “*Jarayu*” or “*Bachadani*”. Perceptions that cervical cancer was not as common as other types of cancer was considered by some women as a contributing factor to this lack of awareness. They related it to their relatively limited understanding of cancer in general:

*“* *I have heard of breast cancer, just the word cancer but have not heard beyond this that what types of cancer are there.* *I just know that cancer is a disease, it occurs and is not very good. A person can die of it. I don’t know anything else about it.”*

(Participant 11)

Of the women who were aware of cervical cancer, some accurately described the relevant anatomical location, whereas others were uncertain of the part of the body involved—*“Not very clear, but I think near vagina”* (Participant 15)—or expressed confusion: *“It means cancer of our reproductive parts like the cervix and the uterus and every part that is related to our reproductive system”* (Participant 4). When asked about other women in the community, the majority of interviewees suggested that lack of awareness was common:

*“No, they have not heard of it either, whomever I have talked to, friends or others don’t know, even I have heard of it today for the first time. We hear that someone died of cancer… but especially this cancer cervical, no one knows.”*

(Participant 7)

It was found that low knowledge levels were thought to be more pronounced among less educated women—*“The women that are highly educated know about it”* (Participant 1)—those not working in health care*—“Only the people working in GP clinic or who see lots of people coming with cervical cancer or cancer treatment and things and all, but rest are not that aware”* (Participant 14)—among the older generation—*“I think younger women are more aware, not the older, like my mum’s, not as much.”* (Participant 19)—and women from rural backgrounds—*“It also seems that some people that come from backward areas [rural] in our country may not know”* (Participant 6). Women who knew of cervical cancer expressed that it was not something prevalent in Asian women:

*“Living in this society here, I have noted, that common women think that it is more of white people thing, women living here get it due to having different sexual partners. And that is why government has a rule that you need to get Pap smear once a year.”*

(Participant 5)

2. Understanding of cervical cancer risk factors is lacking or incorrect:

Confusions about the underlying causes and potential risk factors for cervical cancer were evident among the participants. A common notion was that this cancer is hereditary in nature:

*“Women do not have awareness that it can be caused by sexual partners, their general perception is that it is a genetic disease, hereditary, it is a transmitted from mother or father’s side of the family.”*

(Participant 2)

Other misconceptions on risk factors included dietary and nutritional deficiencies, lack of proper and adequate personal hygiene, trauma and mishandling during childbirth, alcohol abuse, smoking, and infections of female reproductive organs. Some participants could associate risk of cervical cancer with being “*sexually active*” (Participant 9), and believed that “*multiple sexual partners*” (Participant 16) was a major contributing risk factor. Few could relate more to symptoms of the disease rather than risk factors—*“I* *cannot think of any cause that can lead to it. I do know of symptoms.”* (Participant 5)

All but one of the participants were unaware of the link between human papilloma virus (HPV) and cervical cancer. When questioned to recall, only three “*have heard of it [HPV], but [they] have no idea about it*” (Participant 10). Confusion of HPV with other viruses was also present—“*I think there is …. hepatitis C, so it is for that*” (Participant 6). The individual who was aware of its relation to cervical cancer explained it as follows:

*“You get infected with that virus, eventually it gets built up, and that can slowly, like grow over there, it can get into a tumor and slowly it can turn into cancer over time.”*

(Participant 17)

When specifically asked about HPV vaccine, only one participant had been vaccinated and had received it in their native country. The majority of participants had no information and were unaware that a vaccine could prevent cancer:

*“I did not know that cervical cancer can be prevented by vaccination too.”*

(Participant 15)

3. Comprehension of the cervical screening test’s true purpose:

Participants could recall the term “Pap test” more commonly than the term “cervical cancer”.

*“Maybe they would know, just like me, as I know that there is a Pap test, that ladies should get every 5 years and it stops this disease, so they should get it done. I think they would know more of the term Pap smear than cervical cancer.”*

(Participant 6)

When asked how they came to know of the test, the most commonly reported resources were either GP or friends. Women informed that they came to know of it after arriving in Australia and the majority had become familiar with it after giving birth. While many participants had heard of and had had a Pap test in the past, and understood that it was a “*swab of cervix*” (Participant 4), its purpose was not clear among the majority:

*“It’s done inside your vagina, just to see any abnormalities, anything, if you have sort of discomfort and irregular periods, hormonal issues.”*

(Participant 19)

There was also limited understanding of the recommended age prerequisites and screening intervals for the test, with a range of answers including once every year, two years, or five years. Participants expressed that they did not know if the test prevented them from cancers of other reproductive parts. Another misconception was that there is no need for a test in the future if done once with normal results, neither if vaccinated:

*“I got it done 2 times before, everything was okay, and also had vaccine so it was in my brain that I don’t need it.”*

(Participant 19)

Furthermore, only one participant was aware of the changes introduced in the renewed program—*“name changed from Pap to cervical something test”* (Participant 16)—who also expressed sense of ease about the renewed program’s change in screening interval: *“it was a big motivation for me, a point of carefreeness, do it once and forget it for 5 years.”* Only one participant was aware of the option of self-sampling.

**Theme 2:**  **Barriers influencing cervical screening uptake**

Five subthemes related to barriers towards cervical screening participation were identified:

1. Inbuilt convictions about preventive health:

The concept of preventive health action was unfamiliar to participants, who used health care services only when unwell. It emerged that South Asian women do not place importance on screening tests; sexual and reproductive health tests are taken only when women suffer from infections or are pregnant:

*“I think the common reason is why go for a test when we are doing well. Girls think that there is nothing wrong apparently, they have no issue, so they don’t need it. Like I said that if there is no symptom such as bleeding or pain, we think we are all good.”*

(Participant 12)

Fatalistic beliefs, such as fixed time for death, attribution of authority towards God for the disease, and preference for not getting stressed before death, were expressed by some women. Another notion was that women might be afraid of finding about the disease of incurable nature, the fear thus preventing them from taking any tests:

*“There is a time for death, and you have to die once whatever the reason is, why be distressed before it. So, it is the mindset not to know anything bad before it happens.”*

(Participant 10)

Misconceptions on cervical cancer aetiology, such as non-existent family history for this cancer or involvement with multiple partners, resulted in lack of risk perception leading to the question: *“Do I actually need it?”* (Participant 17). Participants elaborated that inborn fear among women, of the procedure being painful, supplemented by the invasive and sensitive nature of the test, could be a reason for lack of attendance.

*“When I was told by the doctor, I was also hesitant how the test will be like, will there be any pain, I had some concerns, So it seems to me that the rest of the women would have the same concerns as mine like if it is painful or not.”*

(Participant 2)

Whereas previous uncomfortable experience was recalled as “*Very distressing, you know the instrument they use for checking was very awful, and it caused me pain as well.*” (Participant 5).

2. Role of healthcare providers and the healthcare system:

Women relied on being externally motivated, commonly by their GP, but most participants reported inadequate information provision by the GPs with focus on the presenting complaint only, rather than opportunistic discussion of cervical cancer prevention. Women attributed it to lack of time spent by GPs or motivation offered to the patients:

*“When you go to the doctor with a specific problem or disease, they just treat that or tell you about that only, not anything more than that. My personal experience is that my GP just asked if I had had it or not, she did not seem interested, or took any action. did not provide any information, and even when I visit, she doesn’t stress about doing it.”*

(Participant 9)

Women also indicated that detailed discussion on gynaecological cancer topics and cervical screening test was mostly provided by their obstetric and gynaecological specialists, and women rarely visited them. Women suggested that South Asian women prefer to visit native language-speaking doctors “*that they can talk easily to and feel comfortable with*” (Participant 2); however, these doctors may not recommend screening as strongly as “*Australian*” doctors.

Women spoke about differences in the health care system between their country of origin and Australia, elaborating that health care practitioners only recommend tests when patients present with severe ailments. Cervical screening is not commonly offered and the majority of the population that is uneducated blindly accepts what the doctor chooses:

*“In my country I have noted that women conceive, even they give birth, but doctors do not tell them nor do they have enough information that Pap test is such a test that diagnoses cancer. Only when one who has some kind of problem, like lots of bleeding then the doctor would say to do the test. And when your doctor recommends it, only then you think about and do it.”*

(Participant 15)

High cost of the diagnostic test was also considered as an important cause of not getting the tests done in the native country. It was further explained that if such tests would not be covered in Australia, women would not have them. Another factor related to the Australian heath care system was the ineffective reminder system, with women complaining of not getting timely reminders: *“When I came to Australia, I got lots of letters, but for the last few years I did not get any letter from the GP.”* (Participant 3). It was also found that for recently arrived immigrants, navigating the health care system could be challenging, since women might not be aware of the available facilities. One participant who was fairly new and had arrived a year ago stated:

*“I don’t know much, I am new from India, have not been to the doctor yet.”*

(Participant 11)

3. Practical constraints:

While many women had basic English skills, understanding more complicated topics or scientific jargon was indicated as a significant barrier. Most participants preferred to receive health care in their own language:

*“Even though they understand as they are educated, sometimes there are names of diseases that they won’t even know about. For example, I have studied accounting, I do not understand all medical terms, if I do NAATI [stands for National Accreditation Authority for Translators and Interpreters] course I might come to know but not now. So, if you are educated, uneducated whatever, the language plays a very important role in making people understand in a better way.”*

(Participant 6)

Lack of time was a frequently projected hurdle in seeking screening, often faced due to childcare responsibilities and household chores:

*“I myself do not have any problem of shyness or anything, even got the report written two times, but could not go because of lack of time, something comes in between like about the child. And after that you even forget about it.”*

(Participant 10)

South Asian women’s dependence on their husband for transport and finance was another important factor brought up by participants. This dependence was more pronounced for new immigrants, not covered by Medicare:

*“Then if they cannot drive, they have to wait for husbands, who are busy in the week, so they wait for the weekends, and then usually doctors are not available on the weekends, these are factors that certainly delay this thing. Then some of the ladies, they are not working they need money, and then they need to depend on the husband for the money.”*

(Participant 4)

It further emerged that women with low literacy and English language proficiency levels were also dependent on their husbands for interpretation of what the doctor said during the visit, especially if the doctor was not native language speaking.

4. Societal and cultural norms influencing decisions and uptake:

Stigma was commonly attached to discussing sexual health issues, with women expressing discomfort in talking about cancer or tests of such intimate nature in public. It was linked to the values and upbringing prevalent in South Asian culture. Participants explained that women were reluctant to discuss such taboo topics openly even with close friends or relatives:

*“We discuss such problems only after marriage, such as ladies’ problems, at most we ask our mothers, but they don’t tell in detail that these problems can be there in women.”*

(Participant 14)

Based on these sociocultural values, modesty was identified as a main barrier towards seeking cervical screening, with women disliking showing private parts without need to anyone. Participants indicated preference for female health care providers among women in the community, but few explained that women might be ashamed to get the test done by female practitioners as well:

*“Certainly, modesty really is a problem, this is the main point to be noticed. The doctor does suggest, but girls maybe because of shyness do not pursue this much. Embarrassment plays a role even more than awareness.”*

(Participant 1)

Participants elaborated that, since some women believe that this cancer is a result of promiscuity or involvement in bad deeds, it leads to imperceptive behaviours, suggesting they are not at risk of the disease:

*“Because in our culture it is thought that if you marry you won’t have any problems, such problems occur when you do things out of marriage. I think that is the reason if they are married, they consider they won’t have this cancer or infections.”*

(Participant 15)

Interestingly, faith was not viewed as a barrier in accessing cervical screening, as it encouraged people to ensure health care. Another barrier raised by participants was lack of autonomy or independent decision making, prevalent among South Asian women. According to the participants, women in South Asian cultures feel reluctant in taking health decisions on their own, and usually involve spouses, parents, or in-laws. This, suggests possible influence of partners’ views on screening uptake:

*“There could be some that think that aah I don’t think you need that thing. Are you having any problem, if you are not, why do you bother doing it?”*

(Participant 17)

However, other women explained supportive nature of spouses also: *“They seem encouraging mostly, like whatever test you want, you can get it done”* (Participant 9). Another cultural imperative provided by the women was that South Asian women do not give priority to their health, but with increasing time in Australia, this attitude may be changing:

*“I would give preference to my son, even in sickness, even if I am tired, do that for my son, my husband, my family, you know. But I can see a difference in younger generation, They look after their kids, but they look after themselves too at the same time.”*

(Participant 5)

**Theme 3:**  **Increasing Cervical Screening Participation**

Participants provided valuable insights on how to best increase participation in cervical screening within their ethnic group.

1. Provision of appropriate information needed:

Most of the women were of the view that detailed information about various aspects of cervical cancer prevention is required. They stressed on the need to address inadequate information on risk factors for the disease:

*“Like how this is caused, what happens, what are the signs and how can we stop it. Obviously, most of it has to do with intercourse, sexual activity, but there are other causes too, so we need to inform about that.”*

(Participant 11)

They indicated that it would be useful to explain the nature of the disease—*“like sometimes you can’t have symptoms and still get it”* (Participant 16)—its prevalence in South Asian countries, and its prevention through the screening test. They also stressed on the need for clearer instructions on when to undergo testing with special reference to age and screening interval changes introduced in the renewed cervical screening program—*“This new thing as you said about self-testing, people do not have any idea about it, telling them is important.”* (Participant 5)

Women expressed that the information can be better understood if provided in their native South Asian languages especially for first generation migrants and refugees, unable to comprehend English:

*“Those who understand English, it is okay to relay in English, but majority, say at least half of the women are those who understand better in Urdu, if it is only in English, they won’t be able to understand. It is necessary that the information is in all languages. It is difficult to explain things sometimes, and because most of community used to come through hardworking. These days immigrants are different they can understand but still some come through deport and are refugees they do not have a lot of English background.”*

(Participant 14)

2. Proactive health care workers and health system’s change facilitate access:

Participants had strong opinion that it would be beneficial if health care professionals were more proactive in informing and encouraging their patients about cervical screening. According to them, GPs are in the best position as they are held in high esteem by their patients, and if motivated by them, it would decrease reluctance shown by women.

*“Doctors are given quite importance in our community, people follow them, So, if GP tells during the routine check-up about what it is and why the test is done, it will be taken seriously.”*

(Participant 13)

They also highlighted that GPs from South Asian background, particularly visited by the women, are considered trustworthy, and can alleviate any concerns, fears, and misconceptions that women may hold regarding the screening procedure.

*“Yes positively, because usually the people come to them because the GP can speak the same language, such as Afghanis and many Urdu speaking people, they come solely for this reason that they do not have to face any barrier while speaking their language, and GP can convince them quite effectively too. So, GPs should be asked, that whenever the women, especially those that cannot understand English language, come to them they should talk to them about it. In fact, GPs can be the best source to convince them.”*

(Participant 2)

Moreover, women elaborated that, since South Asian culture places emphasis on seeking a female health care provider for such intimate procedures, it would be helpful if capacity of female doctors providing the test could be built, or male doctors could make arrangements to refer their patients to female doctors. Other suggestions offered by the participants to increase uptake among women, were to send out reminders and make the test standard rather than optional:

*“Every couple of months, like every 6 months, when you do a full blood count and your functional tests, if you make it just a standard procedure, that you have to get this test done. And explain to the women that this needs to be done, it is like a standard procedure.”*

(Participant 20)

3. Availability of self-sampling—a convenient option:

Almost all participants expressed a positive and encouraging attitude towards the new HPV self-sampling method. According to them, this method would overcome barriers present due to modesty:

*“This change is very helpful in routine testing; women will do the test more willingly if allowed to do in their comfort instead of getting it done by a GP. It is such good news that they could do it because definitely there is an element of modesty.”*

(Participant 13)

It was also suggested to be a convenient option for those women unable to do so because of religious barriers or facing fear and lack of time. Although the participants proposed to make the test readily available, they stressed that proper directions need to be provided:

*“But for that there is a need to create awareness as well as directions on how to carry out the test in the right way. For example, like we do our pregnancy test, we know beforehand that two lines will appear. So, it will be good if shown in a better way.”*

(Participant 12)

Reservations were also expressed by some women whether they could perform the test accurately and some preferred to have the test done by a GP:

*“I think I would not be able to take the liquid or whatever is required in the right way. I think the doctor can take it according to the required way.”*

(Participant 11)

4. Proper channels could be helpful in dissemination of screening information:

Women held various views on different venues that could be used for dissemination of knowledge including GP visits, social media, workplaces, and informal social gatherings. It was also indicated that videos (that can be sent via portals like WhatsApp, Facebook, and Youtube) might be more beneficial than brochures, because *“The spoken words or face to face interaction have more effect, people do not have time to read”* (Participant 15). Additionally, targeted media campaigns through social media could be potentially useful:

*“You know like Queensland government has forced advertisements, they could just create one for spreading the awareness of importance of having Pap test and cervical screening, just as they do for you know smoking, and they declare smoking is injurious for health, just keep women informed about it.”*

(Participant 16)

Another interesting portal suggested by one participant was use of products for menstrual hygiene to display the information. Furthermore, participants expressed that informative talks at schools would also be possibly helpful. However, the use of specific individualised strategies for the underreached groups was stressed:

*“I think that the people who are already aware, and who are getting the test already will get it done if they are reminded about it through the text messages. But the ones that have no awareness of it would not benefit from mobile messages, they will go through them and delete them. By telling them this short sentence: ‘Take the cervical screening test’, will not be effective enough for them. To move such people the best way can be door to door visit, talk with them in their own languages, make them understand and convince them.”*

(Participant 3)

5. Addressing behavioural and societal barriers:

Participants elaborated that information provision needs to be in a manner to address lack of risk perception and health prioritisation among women. According to them, messages such as *“It is the matter of safety, so before the cancer comes at that stage, get this test done”* (Participant 7) and *“if I am happy and healthy, I can take care of my husband and kids. I need to think about myself as well”* (Participant 17) would help women understand its importance. Women explained that discussing the topic among friends and peers would create normalisation, thus influencing health-seeking behaviours indirectly, as described by Participant 16:

*“Since there is a real quick impact on our woman that oh, she got it so I should get it too. Not in a bad way but there is something in our culture and society that we tend to keep up with the others, so I think just talking about it when sitting together and socializing, bringing out the topic and saying that it is just like a blood test, nothing bad or thing to look down on.”*

(Participant 16)

Women were also of the view that sociocultural values resulting from partner influence could be better addressed by involving spouses and explaining the importance of the procedure to them. One participant further elaborated that a better way to do this could be as follows:

*“Awareness should be given to males as well, you know when sometimes husband and wife both go to the GP, bringing it up in front of husband that it’s just a general test.”*

(Participant 2)

Women also stressed the need of breaking free from the cultural notion of reluctance in talking to daughters about sexual health issues. They indicated that it was the need of the hour to keep younger women well informed:

*“Especially parents like mothers need to tell their daughter; mothers, sisters and close friends need to inform others. If a mother won’t tell her child about it, why would another person do it.”*

(Participant 12)

## 4. Discussion

This study provides in-depth understanding of the perspectives and behavioural attitudes that South Asian immigrant women in Australia hold towards cervical cancer and its prevention. The most prominent factors affecting cervical screening uptake included inaccurate and inadequate information deep-rooted in sociocultural beliefs, lack of preventive health concepts, low prioritisation of one’s own health, reliance on GPs for direction, lack of time, role of language and emotional factors, and practical and financial dependence on spouse.

This study found that awareness and knowledge of HPV and cervical cancer was generally low and incorrect, and this affected cervical screening uptake. Lack of proper knowledge has been shown to affect immigrant women’s cervical screening behaviours in other studies as well where women were unable to make the connection between cervical cancer and Pap test [18]. Further exploration of the misconceptions around the topic in our study revealed confusions about cancer site and its risk factors. Similar misconceptions on associated role of diet, exercise, and personal hygiene as risk for cervical cancer were observed in Thai and Chinese women [16,31]. While cultural concepts around supernatural powers affecting health were highlighted in African women [19], such cultural perspectives were not mentioned by South Asian women in our study. Instead, they linked cervical cancer to having multiple sexual encounters, which, according to their cultural understanding, is not common among South Asian women, thus leading to lack of risk perception. Inability to delineate the link between HPV and cervical cancer, as evident in this study, is also consistent with the findings in previous studies [32,33]. Additionally, a preceding quantitative survey has also shown lack of HPV and cervical cancer knowledge among South Asian women [24]. Informative interventions for immigrant populations could be impactful if special attention was paid to address these deficits and clarify specific misconceptions, thus paving a way towards greater clarity over what cervical cancer is and its risk factors are.

With the changes in the renewed National Cervical Screening Program, this study was also novel in assessing immigrant women’s views on the self-sampling HPV test method. It appears that there is still limited awareness on this option among women. Concerns of women, such as feeling of anxiety towards accurate performance of the test, along with that of convenience, raised in our study are similar to the ones elaborated in a previous qualitative study exploring perspectives of native Australian women on changes made in the renewed program [34]. Thus, steps are required to increase dissemination of information about the current cervical screening program recommendation and the availability of self-collection among never and infrequent testers. Ideally, this information needs to be made available in languages comprehended by these respective women, with special emphasis on easy-to-follow directions for the self-sampling test procedure.

This study emphasises the importance of the role that can be played by GPs, to persuade South Asian immigrant women of the importance of cervical screening, since all previous studies involving immigrant women in Australia stress the valued aspect of HCP support [19,31]. South Asian women prefer female doctors who can speak their first language as they can relate to their problems in a better way. Our study suggests that there is a marked difference in concepts of seeking health care services among South Asian immigrants, which results from lack of patient involvement in decision making and emphasis on diagnostic rather than preventative tests due to symptom absence, prevalent in their native countries. However, what HCPs think, and what barriers they face while dealing with South Asian women in Australia, remains unknown, and needs to be researched. Despite this, enthusiastic involvement of GPs can increase cervical screening participation among immigrant women, as suggested by an interventional study in Norway [35].

Cultural barriers, such as lack of health prioritisation, dependence on partners, and modesty, as found in our study, are similar to those that have been reported in previous studies involving South Asian women in other countries [15,36,37,38]. Similarly, the self-sacrificing nature of the women, along with sole responsibility for domestic chores, as indicated in this study, leads to a lack of time for oneself, thus affecting health-seeking behaviours, as reported in another systematic literature review involving South Asian women [39]. With literature pointing towards predominant patriarchal roles in South Asian households [40,41], this hurdle needs to be considered, developing ways to improve partner advocacy, or to reach women without being defendant on partners for health care services.

An important point to consider is the acknowledgement of immigrant women groups at high risk of missing important cervical cancer screening examinations, including those that have recently arrived, from rural backgrounds, asylum seekers, refugees, or those unable to comprehend English. Targeted interventions for these groups might be the key to success in overcoming the cervical screening disparities.

Authors should discuss the results and how they can be interpreted from the perspective of previous studies and of the working hypotheses. The findings and their implications should be discussed in the broadest context possible. Future research directions may also be highlighted.

## 5. Conclusions

This study provided rich and detailed insights on the views of Australian South Asian immigrant women surrounding cervical screening. It aimed to capture the voice of less educated South Asian immigrant women towards cervical cancer prevention. It highlights the importance of using an evidence-informed approach, to introduce culturally appropriate initiatives to increase screening participation among currently underreached immigrant populations.

## Figures and Tables

**Table 1:** Characteristics of 20 participants in the study.

Characteristic	Number of Participants (*n*)
Age	
20–30 years	8
31–40 years	6
41–60 years	6
Language	
English	5
Other (Urdu, Hindi, Punjabi, Fijian Indian)	15
No. of children	
None	2
Two or less	9
Three or more	9
Ethnicity	
Indian	9
Pakistani	6
Other (Bangladeshi, Nepalese, Sri Lankan, Indian Fijian)	5
Education completed	
School (Secondary)	15
University (Undergraduate)	5
Duration of stay in Australia	
Less than 5 years	3
5–10 years	14
More than 10 years	3
Cervical screening participation	
Yes—Up to date	8
Yes—Not up to date	6
No	6

## Data Availability

Not applicable.
